# Improvement of thermostability and catalytic efficiency of xylanase from *Myceliophthora thermophilar* by N-terminal and C-terminal truncation

**DOI:** 10.3389/fmicb.2024.1385329

**Published:** 2024-04-10

**Authors:** Yue Yang, Chengnan Zhang, Hongyun Lu, QiuHua Wu, Yanfang Wu, Weiwei Li, Xiuting Li

**Affiliations:** ^1^Key Laboratory of Geriatric Nutrition and Health (Beijing Technology and Business University), Ministry of Education, Beijing, China; ^2^Beijing Engineering and Technology Research Center of Food Additives, Beijing Technology and Business University (BTBU), Beijing, China; ^3^Department of Exercise Biochemistry, Exercise Science School, Beijing Sport University, Beijing, China

**Keywords:** GH11 xylanase, N-terminal and C-terminal truncation, thermostability, catalytic efficiency, intrinsic mechanism

## Abstract

**Introduction:**

Extracting xylanase from thermophilic filamentous fungi is a feasible way to obtain xylanase with good thermal stability.

**Methods:**

The transcriptomic data of *Myceliophthora thermophilic destructive* ATCC42464 were differentially expressed and enriched. By comparing the sequences of Mtxylan2 and more than 10 xylanases, the N-terminal and C-terminal of Mtxylan2 were truncated, and three mutants 28N, 28C and 28NC were constructed.

**Results and discussion:**

GH11 xylan Mtxylan2 was identified by transcriptomic analysis, the specific enzyme activity of Mtxylan2 was 104.67 U/mg, and the optimal temperature was 65°C. Molecular modification of Mtxylan2 showed that the catalytic activity of the mutants was enhanced. Among them, the catalytic activity of 28C was increased by 9.3 times, the optimal temperature was increased by 5°C, and the residual enzyme activity remained above 80% after 30 min at 50–65°C, indicating that redundant C-terminal truncation can improve the thermal stability and catalytic performance of GH11 xylanase.

## 1 Introduction

Xylanase (EC 3.2.1.8) degrades xylan by stoichiometric hydrolysis of the β-1,4-glycosidic bond to produce oligosaccharides of different lengths (Shi et al., 2013). Based on structural similarities, xylanases are categorized into different families (Paes et al., 2012). Among them, GH11 xylanase has a conserved β-jelly roll structure with a right-handed partially closed conformation (Paes et al., 2012). GH11 xylanase has a small molecular weight, fast catalytic efficiency, and excellent substrate selectivity (Li C. et al., 2018; Teng et al., 2019; Wang et al., 2021). These properties make GH11 xylanase promising for a wide range of applications in industries such as animal feeding, food baking, and pulp bleaching. However, in order to maximize production efficiency, these industrial processes often need to be carried out at high temperatures, which places high demands on the heat resistance of GH11 xylanase. For example, in the pulp bleaching process, the use of xylanase at high temperatures and alkaline pH conditions improves the permeability of bleaching chemicals, thereby minimizing the process flow (Wang et al., 2016). Adding xylanase without cooling down during the production of animal feed can reduce the production time of the pelleting process (Muhammad et al., 2018). The addition of xylanase to raw materials during high-temperature saccharification can lead to higher production rates and a lower risk of contamination (Turner et al., 2007). However, the poor thermostability of most natural GH11 xylanases struggles to meet industrial demands (Badieyan et al., 2012; Zhang et al., 2014; Robledo et al., 2016; Han et al., 2019). Therefore, enhancing the thermostability of GH11 xylanase has become a critical bottleneck to be solved (Xing et al., 2021; Wu et al., 2023).

Mining of xylanases with strong thermotolerance from *Myceliophthora thermophilic destructive* has been shown to be a viable pathway (van Gool et al., 2013). *Thermophilic destructive* fungus is a thermophilic filamentous fungus derived from soil (van Gool et al., 2013; Berezina et al., 2017). It was found to secrete a variety of heat-stable xylanases and cellulases that efficiently hydrolyze biomass such as hemicellulose and cellulose (Karnaouri et al., 2014; Singh et al., 2016; Dos Santos Gomes et al., 2019; Anu et al., 2022). Xylanases from *Thermophilic destructive* fungus xylanase have been reported to be thermally stable at 30–50°C (van Gool et al., 2013; Boonrung et al., 2016; Katsimpouras et al., 2019; Liew et al., 2019). However, two new GH11 xylanase genes, *MYCTH_56237* and *MYCTH_49824*, were cloned from the *Myceliophthora thermophilic destructive*, which also has the ability to hydrolyze maize kernels better at 60–70°C (Basit et al., 2018). A study on xylanase secreted by *Thermophilic destructive* fungus found that overexpression of transcription factors can result in an increase in xylanase production, improving the catabolism of corncobs (Wang et al., 2015). However, the mining of *Thermophilic destructive* fungus-derived xylanases still needs to be carried out intensively. The wide application of transcriptomics techniques in the screening of microbial-derived enzymes has led to the reporting of abundant data on biological samples. Deeper mining of these data can be more targeted and efficient to find target enzymes produced by microorganisms with excellent properties, which has been the basis of many important research discoveries (Wang and Xia, 2008; Wang et al., 2018; Liu et al., 2022).

Another strategy to enhance the thermostability of GH11 xylanase is rational modification. Numerous studies have found that structural changes of xylanases in high-temperature environment often start from the N-terminal or C-terminal. Wang and Xia (2008) constructed mutants with significantly higher thermostability by exchanging homologous fragments of the C-terminal ends of two xylanases, XlnB and XlnC, both from *Streptomyces sanguinis*. Similarly, thermal stability derived from *Aspergillus oryzae* can be improved by substituting N-terminal residues (Yin et al., 2013). Therefore, rational modification for the flexible N-terminus or C-terminus of GH11 xylanase becomes an effective method to improve the thermostability of GH11 xylanase (Han et al., 2018; Kumar et al., 2018; Li Q. et al., 2018; Li et al., 2019; Pan et al., 2022). The rapid development of bioinformatics technology has provided multiple effective means for developing rational modification methods (Ventorim et al., 2018; Min et al., 2021). Comparative analysis with gene sequences of enzymes with higher thermostability and prediction of flexible regions using molecular modeling methods have all been widely shown to be reliable methods for developing xylanase modification strategies (Liu and Wang, 2003; Wang et al., 2016; Cayetano-Cruz et al., 2021; Li et al., 2023).

In this study, we first identified *Mtxylan2*, a xylanase gene with potential heat-resistant properties, by performing differential expression analysis as well as enrichment analysis based on the transcriptomics data of *Myceliophthora ATCC42464*, a source published in the public database. Subsequently, it was rationally modified for the N-terminus and C-terminus by using bioinformatic methods. The obtained mutant 28C showed a 9.3-fold increase in catalytic activity, the optimal temperature was increased by 5°C to 70°C, and the residual enzyme activity could still maintain more than 80% after holding at 50–65°C for 30 min, which has potential application value and development prospects in industrial production.

## 2 Materials and methods

### 2.1 Sequencing data download and processing

The eligible gene data matrix file GSE137286 dataset was retrieved from Gene Expression Omnibus (GEO; https://www.ncbi.nlm.nih.gov/geo). Samples with xylose as substrate were selected: GSM4074520, GSM4074530, GSM4074539, and GSM4074540. Sample types were categorized as wild-type WT and deletion-type DXyr1.

### 2.2 Differential expression analysis

After downloading the gene matrix data, it was processed using Perl 5.32.1 and R 4.0.5 software and R packages such as limma, ggplot, and clusterProfiler. Differentially expressed genes (DEGs) were screened by background correction, normalization, and expression value calculation, and *p* < 0.05 and logFC >0.5 [fold change (FC)] were used as the screening criteria for the differentially expressed genes, with a positive logFC value representing upregulation and a negative logFC value representing downregulation. A positive logFC value represents upregulation of expression, and a negative value represents downregulation of expression. The screening of differential genes was demonstrated by volcano plots. Two R packages, ggplot2 and ggrepel, were used to draw the volcano diagram.

### 2.3 Enrichment analysis

The screened up- and downregulated DEGs were analyzed for genomic database (KEGG) and gene ontology (GO) enrichment using the David database, the online tool KOBAS 3.0, and the R package clusterProfiler (v 3.10.1), respectively. The obtained signaling pathways were screened by a set threshold value of *p* < 0.05, and the online tool was used to draw GO histograms, with the horizontal coordinate indicating the degree of their enrichment correlation and the vertical coordinate for each GO, which includes biological process (BP), cellular component (CC), and molecular function (MF).

### 2.4 PPI network analysis

PPI protein networks were established using the String database. Differential genes were visualized, the size of the degree value was calculated by Cytoscape 3.8.1 software and the CytoNCA plug-in, gene correlations were ranked and analyzed, and PPI network maps were plotted.

### 2.5 Selection of key genes

The key genes were sorted by degree value, and the top seven genes with higher differential expression content were selected and compared using seven public databases: non-redundant protein sequence database (Nr), nucleotide sequence database (Nt), Pfam, clusters of orthologous groups for eukaryotic complete genomes (KOG), and Swiss-prot protein sequence database (Swissprot). Nucleotide sequence database (Nt), Pfam, clusters of orthologous groups for eukaryotic complete genomes (KOG), and Swiss-prot protein sequence database (Swissprot) were used to compare the genes, and the key genes were functionally annotated and classified. The key genes were functionally annotated and classified. The key xylanase genes with potential heat resistance and high differential expression content were screened.

### 2.6 Gene, strains, and substrates

*Escherichia coli* DH5α cells were used for cloning, and *E. coli* BL21 (DE3) cells were used for protein expression. Plasmids pMD18-T and pET-28a (+), Taq polymerase, DNA gel extraction kit, isopropyl-β-d-thiogalactopyranoside (IPTG), kanamycin (Kan), and ampicillin (Amp) were purchased from Takara (Tokyo, Japan). Restriction endonucleases, T4 DNA ligase, and Q5^®^ High-Fidelity DNA Polymerase were obtained from NEB Inc. (Ipswich, MA, United States). Bovine serum albumin (BSA) was obtained from Roche (Basilea, Switzerland). Birchwood and oat-spelt xylan were purchased from Sigma-Aldrich (St. Louis, MO, United States). *E. coli* cells that included the gene encoding xylanase were cultivated in Luria-Bertani (LB, 10 g/L peptone, 5 g/L yeast extract, and 10 g/L NaCl) medium for protein overexpression.

### 2.7 Construction of xylanases 28N, 28C, and 28NC

28N and 28C were constructed by truncating the N-terminal sequence (1M-11Q) and the C-terminal sequence (206S-260L) of Mtxylan2, respectively. 28NC was constructed by simultaneously truncating the N-terminal sequence (1M-11Q) and the C-terminal sequence (206S-260L) of Mtxylan2. The Mtxylan2 gene was used as a template, and the mutant gene was constructed by overlap extension PCR, which was performed with primers containing the mutated codons (Supplementary Table S1). The PCR cycling conditions included protein denaturation at 94°C for 5 min, followed by 30 cycles of 94°C for 30 s, 65°C for 30 s, 72°C for 30 s, and an elongation step at 72°C for 10 min Mtxylan2 and the mutant gene were ligated into the pMD18-T vector and transformed into *E. coli* DH5α. DNA sequencing has confirmed that the resulting DNA contains Mtxylan2, 28N, 28C, and 28NC. In addition, Mtxylan2, 28N, 28C, and 28NC were ligated into the pET-28a vector at NcoI and XhoI restriction sites and transformed into *E. coli* BL21 (DE3).

### 2.8 Expression and purification of xylanases

The transformants were inoculated into LB medium containing 40 μg/mL Kan and cultured at 37°C and 200 rpm. IPTG was added to a final concentration of 0.5 mM when the absorbance of the culture at 600 nm reached 0.6–0.8 and the temperature was reduced to 20°C to induce xylanase expression. Cells were harvested by centrifugation at 9,391 × g and 4°C for 5 min, resuspended in 50 mM phosphate buffer (pH 7.5), and fragmented by ultrasonication for 15 min. The crude enzyme solution was harvested by centrifugation at 9,391 × g and 4°C for 10 min.

The target enzyme with a C-terminal (His)6-tag was purified using a His-Tag Ni-affinity column (1 × 10 cm, GE Healthcare, Uppsala, Sweden) equilibrated with 50 mM phosphate buffer (pH 7.5) containing 300 mM NaCl and different concentrations of imidazole (0–500 mM) on an ÄKTA FPLC purification system (GE Healthcare). Unwanted contaminating proteins were eluted by washing the column with 50 mM imidazole, and the target protein was then eluted using 200 mM imidazole. The purity of the enzymes was determined by sodium dodecyl sulfate-polyacrylamide gel electrophoresis (SDS-PAGE) using a 12.5% separating gel and a 4.5% stacking gel, as described by Laemmli (1970). The protein concentrations were determined using a BCA protein assay kit (Thermo Fisher Scientific Inc., Rockford, IL, United States) using BSA as the standard (Li et al., 2017). A standard curve was generated using BSA standards at concentrations of 0–0.5 mg/mL. Two hundred microliters of a BCA working solution was mixed with 20 μL of a suitably diluted sample (or BSA standard) and incubated at 37°C for 30 min. The absorbance at 562 nm was measured using a microplate reader (Thermo Fisher Scientific Inc., Rockford, IL, United States). The protein concentration of the sample was calculated from the standard curve.

### 2.9 Xylanase activity assay

Xylanase activity was analyzed according to the method reported by Bailey et al. (1992). The reaction mixture containing 0.1 mL of a diluted enzyme solution and 0.9 mL of 1.0% (w/v) beechwood xylan in 50 mM citrate buffer (pH 6.0) was incubated at 55°C for 10 min. The released reducing sugar content was evaluated by the 3,5-dinitrosalicylic acid (DNS) method using xylose (X1) as the standard. One unit (U) of xylanase activity was defined as an enzyme releasing 1 μM X1 equivalent reducing sugars from the substrate per minute.

### 2.10 Biochemical characterization of xylanase

The optimal pH of xylanase activity was measured at 37°C using 50 mM citrate buffer (pH 3.0–6.0) and 50 mM phosphate buffer (pH 6.5–8.0). The pH stability of xylanase was determined by incubating the enzyme in buffers over the pH range of 3.0–8.0 for 30 min at 37°C and then measuring the residual enzyme activity. The optimal temperature of xylanase activity was measured in 50 mM citrate buffer (pH 6.0 and pH 6.5) at temperatures ranging from 40°C to 95°C. The thermal stability of wild-type and mutant xylanases was monitored by measuring the residual activity of xylanases after incubation at different temperatures for different times. The half-life (*t*_1/2_60) of xylanase was defined as the time for the enzyme activity to drop to half at 60°C. The half-life (*t*_1/2_60) was measured by plotting the enzyme activity over time and was calculated using *y* = *a* × e^−*kt*^ (where a denotes the initial enzyme activity, *t* denotes time, and *k* denotes the decay constant).

The substrate specificity of xylanase was measured using different cellulose and hemicellulose substrates (1%, w/v) at pH 6.0 and pH 6.5. Released reducing sugar content was determined by the DNS method, as described in section 2.10. The kinetic parameters of xylanase were measured with different concentrations (2.5–30 mg/mL) of beechwood xylan under optimal reaction conditions. The *K*_m_ and *V*_max_ of the enzyme were calculated according to the Michaelis–Menten equation using GraphPad Prism software 5.0 (GraphPad Software Inc., San Diego, CA).

### 2.11 Structural analysis and molecular dynamics simulations

Nucleotide and protein sequences were analyzed using NCBI BLAST. The amino acid sequence of Mtxylan2 was aligned using DNAMAN 8.0. Homology modeling was performed using the GH11 xylanase XlnB2 structure (PDB ID: G2Q913.1.A) as the template and the modeler module of Discovery Studio 2020 (DS2020). PROCHECK^1^ was used for structure validation (Guo et al., 2021).

The interaction force and solvent-accessible surface area (SASA) were calculated to compare differences between wild-type and mutant xylanases. The electrostatic potential of xylanases was calculated by the Adaptive Poisson–Boltzmann Solver (APBS). Molecular docking of xylanase with the substrate xylohexaose was conducted using AutoDock 4.2, and visualization and analysis were performed using PyMol 2.4.1. Hydrogen bonding between subsites of xylanase and xylohexaose was analyzed by DS2020. Molecular dynamics (MD) simulations were performed to further assess the global stability of wild-type XynA and mutant XynAR using GROMACS 4.5.4 at 333 K (60°C) for 100 ns. Water molecules were removed from the Mtxylan2 and 28C models, and then these models were hydrogenated and Kollman charges were added. The gromos54a7 force field was used, and the treated protein was placed inside an SPC water model in a cubic box. Sodium and chloride ions were added to maintain neutrality. Protein-energy minimization was achieved using 1,000 steps from the steepest descent and heated to the target temperature. Then, 100 ps NVT and 100 ps NPT simulations were performed sequentially. The temperature was set to 333 K using the V-rescale thermostat with a coupling constant of 0.1 ps, and the pressure was set at 1 bar using Parrinello–Rahman pressure coupling with a coupling coefficient of 2 ps. Finally, models were simulated for 50 ns, and trajectory data were saved every 2 fs. After MD simulations for 100 ns, the root mean square deviation (RMSD) and the root mean square fluctuation (RMSF) were calculated using gmx rms and gmx rmsf of the GROMACS suite, respectively. For RMSD calculations, the initial structure of Mtxylan2 or 28C was used as the reference structure. For RMSF calculations, the whole trajectory (100 ns) was used to reveal the flexibility of amino acid residues.

## 3 Results and discussion

### 3.1 Differential gene screening

**Figure 1:**
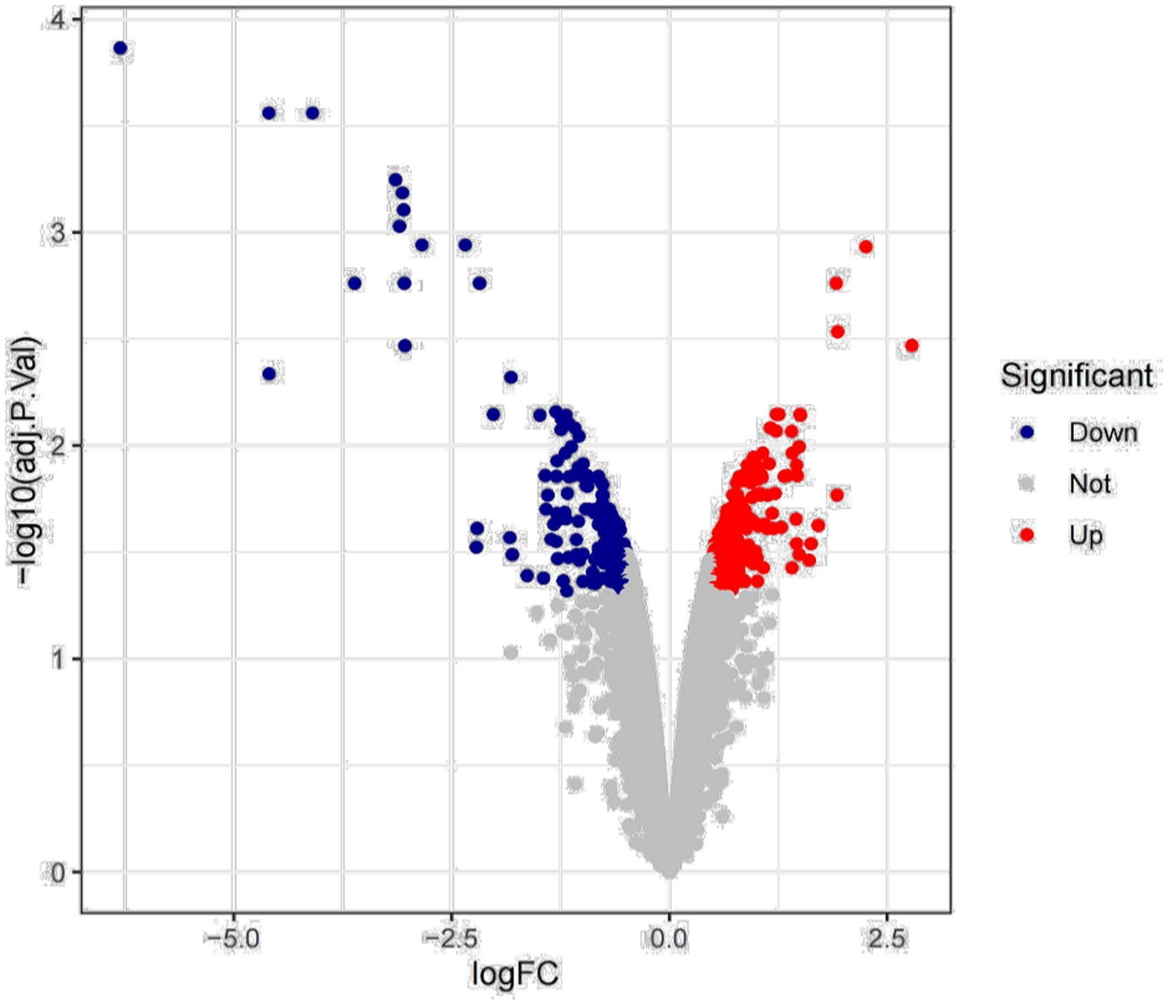
Differential expression analysis.

Previous studies have found that *Myceliophthora thermophila* produces abundant xylanase to hydrolyze corncob, birchwood xylan, and wheat arabinoxylan. Deletion of transcription factors resulted in downregulation of xylanase gene expression, leading to a decrease in endo-xylanase activity (Wang et al., 2015; Dos Santos Gomes et al., 2019). We mined key xylanase genes based on transcriptomic data of wild-type and transcription factor deleted strains grown for 2 h and 8 h with wheat arabinoxylan as substrate. The results of differential expression analysis showed that a total of 416 genes showed changes in expression, of which 244 genes were upregulated and 172 genes were downregulated (Figure 1).

**Figure 2:**
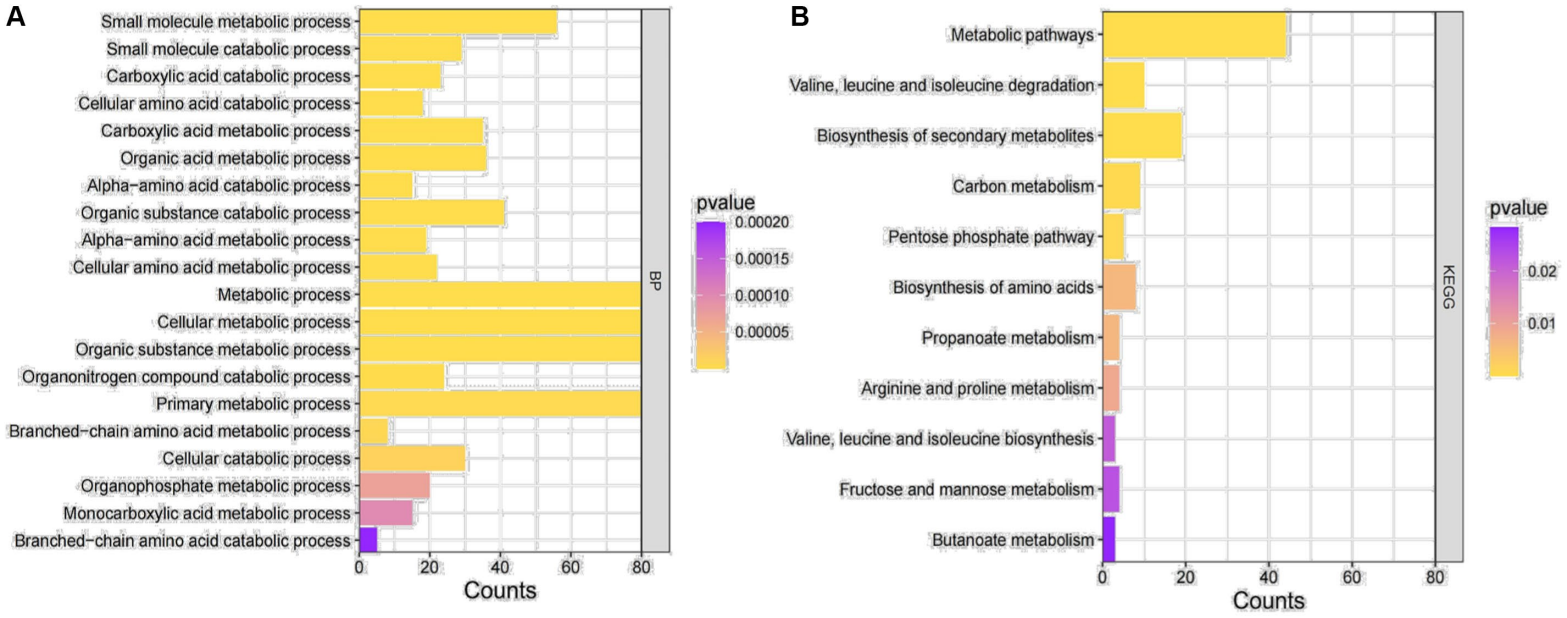
GO enrichment analysis. **(A)** GO functional enrichment analysis of DEGs. **(B)** Enrichment analysis of the KEGG signaling pathway for DEGs.

In order to further explore the biological functions represented by these differential genes and the signaling pathways they are involved in, the differential genes were enriched for GO function and KEGG signaling pathway (Figure 2). Based on the BP pathway enrichment analysis, the differential genes were most strongly associated with “cellular metabolic processes,” “metabolic regulation,” “organic substance metabolic processes,” and “primary metabolic processes” (Figure 2A). In addition, based on KEGG signaling pathway analysis, the genes were mainly enriched in “metabolic pathways” and “biosynthesis of secondary metabolites” (Figure 2B). Therefore, we selected the BP pathway and the KEGG pathway with the highest enrichment intensity for subsequent studies.

### 3.2 Analysis of the PPI protein network

**Figure 3:**
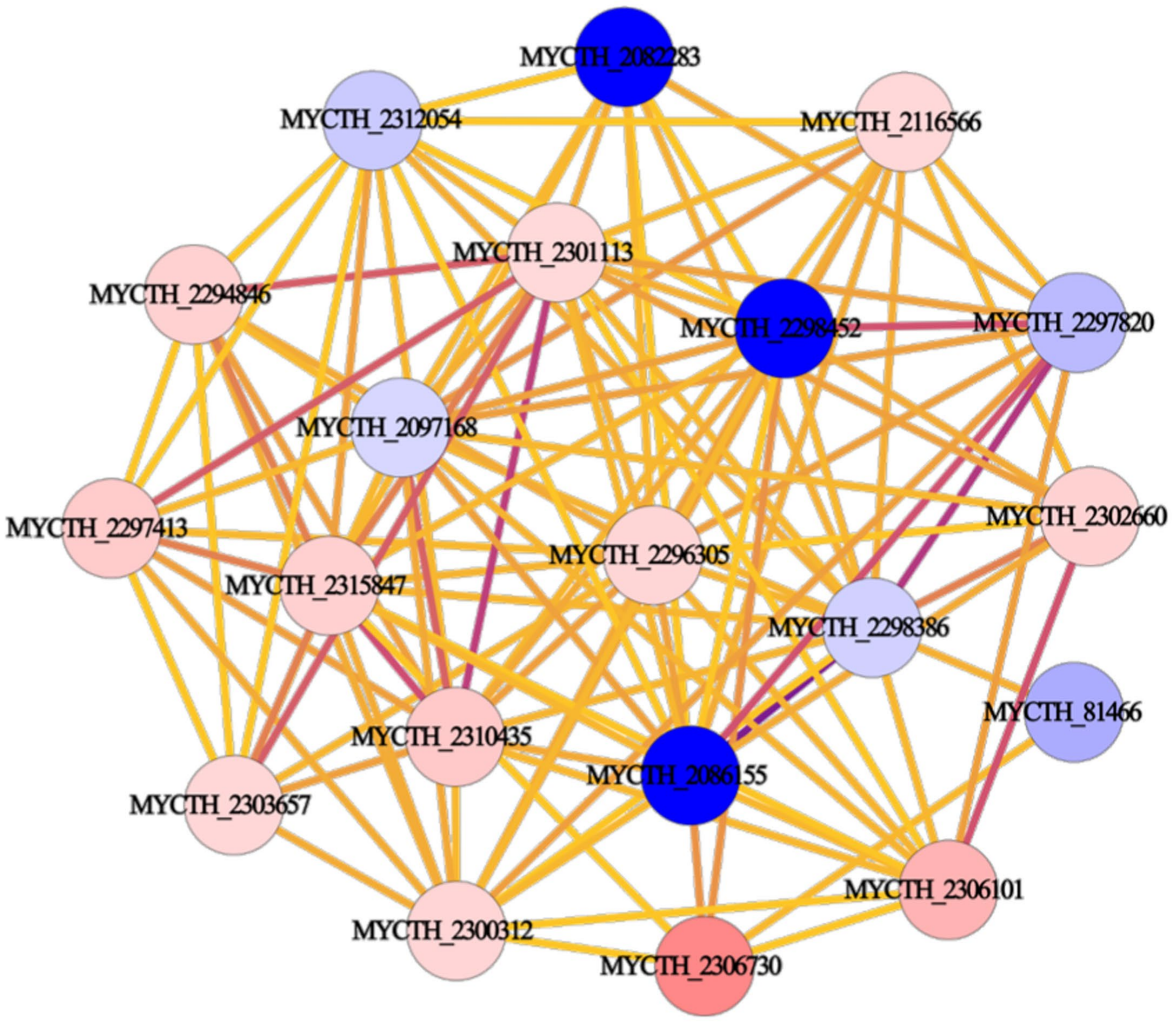
Protein interaction network construction.

We performed PPI network analysis of differential genes in key pathways of “cellular metabolic processes” and “metabolic signaling pathways.” The top 20 highly expressed genes were selected to establish the protein core network, which contains 20 nodes and 115 edges (Figure 3). The key genes represented by 20 nodes were ranked based on degree values and analyzed for their possible functions by gene annotation. As a result, *Mtxylan2* was found to be the xylanase with high differential expression (Supplementary Table S2).

### 3.3 Bioinformatics analysis of the gene Mtxylan2

**Figure 4:**
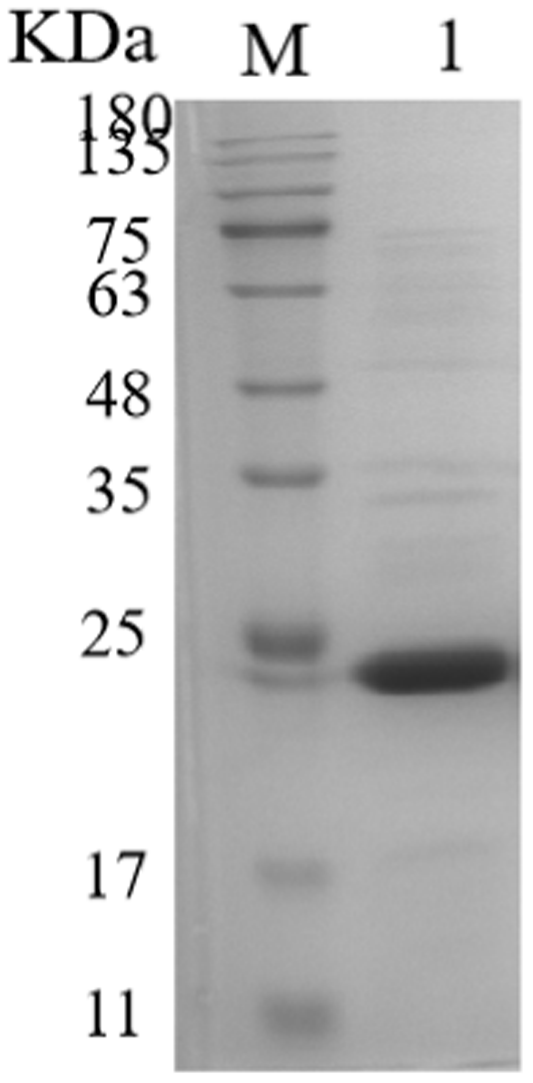
SDS-PAGE analysis of xylanase. Lane M, protein marker; Lane 1, the wild-type xylanase Mtxylan2.

Based on SignalP-5.0 analysis, the *Mtxylan2* gene is 780 bp in length, encodes 260 amino acids, and has no signal peptide sequence at the N-terminus.^2^ The molecular weight and isoelectric point (pI) were estimated to be 25,665.93 Da and pH 7.57, respectively, using ExPASy’s ProtParam tool. A codon adaptation index (CAI) of 0.71 was calculated based on the software, and the GC content was 67.5%. Excessive GC content (>70%) in gene sequences causes increased stability of the RNA secondary structure, slows or suspends translation, and affects gene expression and regulation (Romero et al., 2000; Kudla et al., 2009). Optimization of the Mtxylan2 gene sequence through the http://www.jcat.de/ website reduced its GC content from 67.05 to 55.13%. The codon adaptation index (CAI) was increased from 0.71 to 0.92. SDS-PAGE analysis of purified Mtxylan2 showed that the molecular weight of Mtxylan2 was 25 kDa, which was consistent with the theoretically calculated value (Figure 4).

### 3.4 Biochemical characterization of Mtxylan2

**Figure 5:**
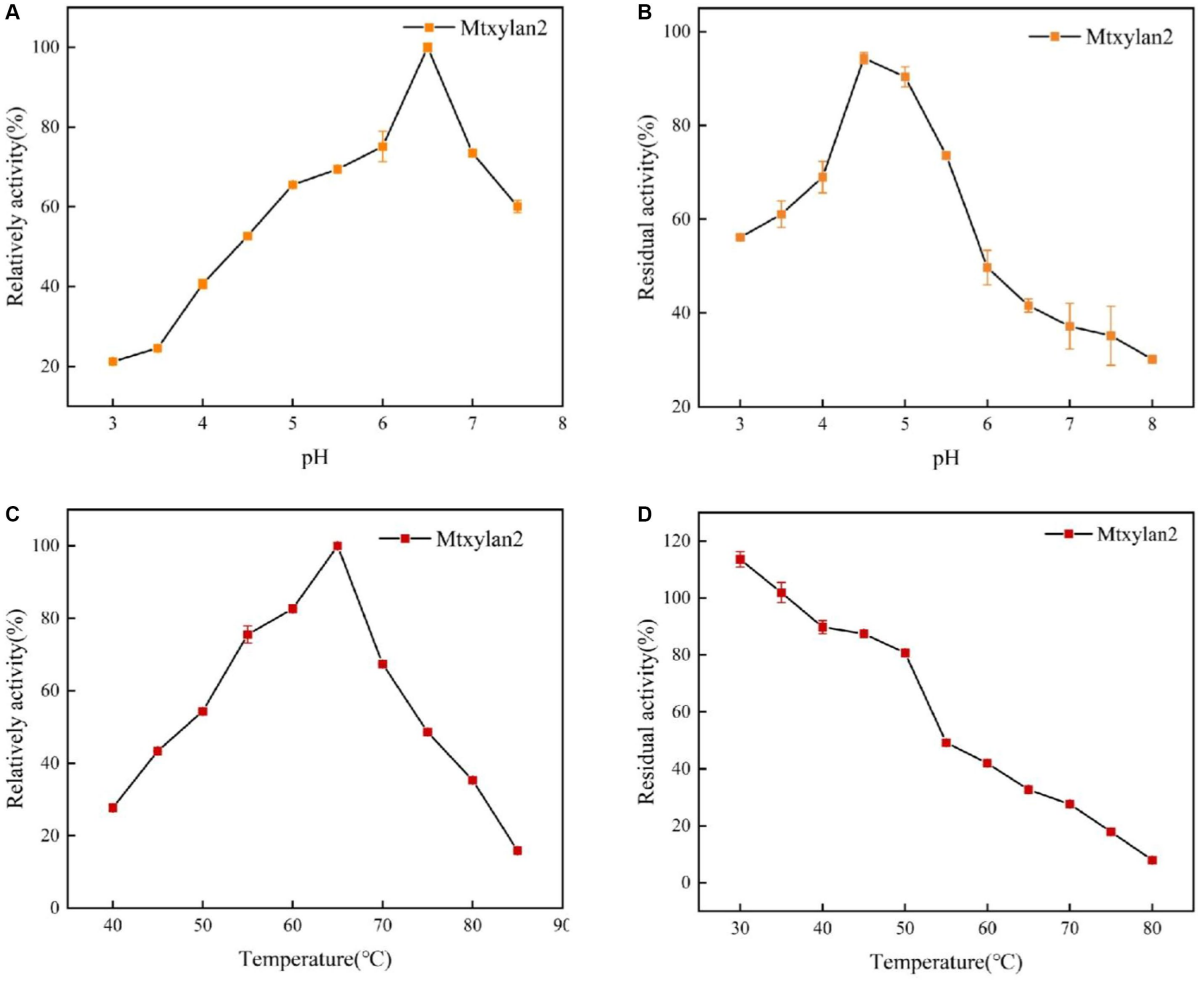
The biochemical characterization of Mtxylan2, 28N, 28C, and 28NC. **(A)** The optimal pH was measured at 50°C for 10 min in different buffers (50 mM) from 3.0 to 8.0; the buffers used were 50 mM citrate buffer (pH 3.0–6.0) and phosphate buffer (pH 6.5–8.0); the highest enzyme activity was used as 100%. **(B)** The pH stability of xylanase was determined in different pH range buffers at 37°C for 30 min, and then the residual activity of the treated enzyme was measured in 50 mM citrate buffer (pH 6.0) at 65°C for 10 min; the activity of untreated xylanase was defined as 100%. **(C)** The optimal temperature was measured in 50 mM citrate buffer (pH 6.5) at different temperatures (40–85°C); the highest enzyme activity was used as 100%. **(D)** Thermostability was determined by incubating at varying temperatures (30–80°C) in 50 mM citrate buffer (pH 6.5) for 30 min; the activity of untreated xylanase was defined as 100%.

The specific enzyme activity, optimum pH, optimum temperature, pH stability, and thermostability of Mtxylan2 were analyzed. It was observed that the specific enzyme activity of Mtxylan2 was 104.67 U/mg (Table 1), determined at 37°C and pH 6.0 using beech wood xylan as substrate, which is lower than other thermophilic xylanase enzymes, limiting its wide application in industry. Measurement of its optimum pH revealed that Mtxylan2 had optimum activity at pH 6.5 (Figure 5A), and the xylanase Mtxylan2 had more than 70% residual enzyme activity in the pH 4.5–5.5 range. However, the residual enzyme activity gradually decreased with increasing pH, and the acidic stability of Mtxylan2 was found to be poor by determining the residual enzyme activity at pH 3.0–4.0 (Figure 5B).

**Table 1:** Specific enzyme activity of polymeric substrates of Mtxylan2, 28N, 28C, and 28NC.

	Substrate specificity (U·mg^−1^)		
Enzyme	Beechwood xylan	Birchwood xylan	Oat-spelt xylan
Mtxylan2	104.67 ± 10.52	32.43 ± 2.94	44.03 ± 4.79
28N	249.96 ± 1.49	231.09 ± 3.33	197.67 ± 2.78
28C	973.74 ± 22.58	867.42 ± 3.97	591.35 ± 23.35
28NC	429.71 ± 27.94	335.15 ± 30.30	347.46 ± 3.03

For the optimum temperature and temperature stability, the optimum temperature for Mtxylan2 was 65°C (Figure 5C). Mtxylan2 could maintain about 80% or more of its residual activity when incubated for 30 min between 30°C and 50°C. However, at temperatures exceeding 50°C, Mtxylan2 showed only minor residual activity (Figure 5D). Mtxylan2 was found to have a higher optimum temperature, lower thermal stability, and acceptable catalytic activity by comparison with the xylanases reported (Supplementary Table S3).

### 3.5 Sequence analysis of Mtxylan2

**Figure 6:**
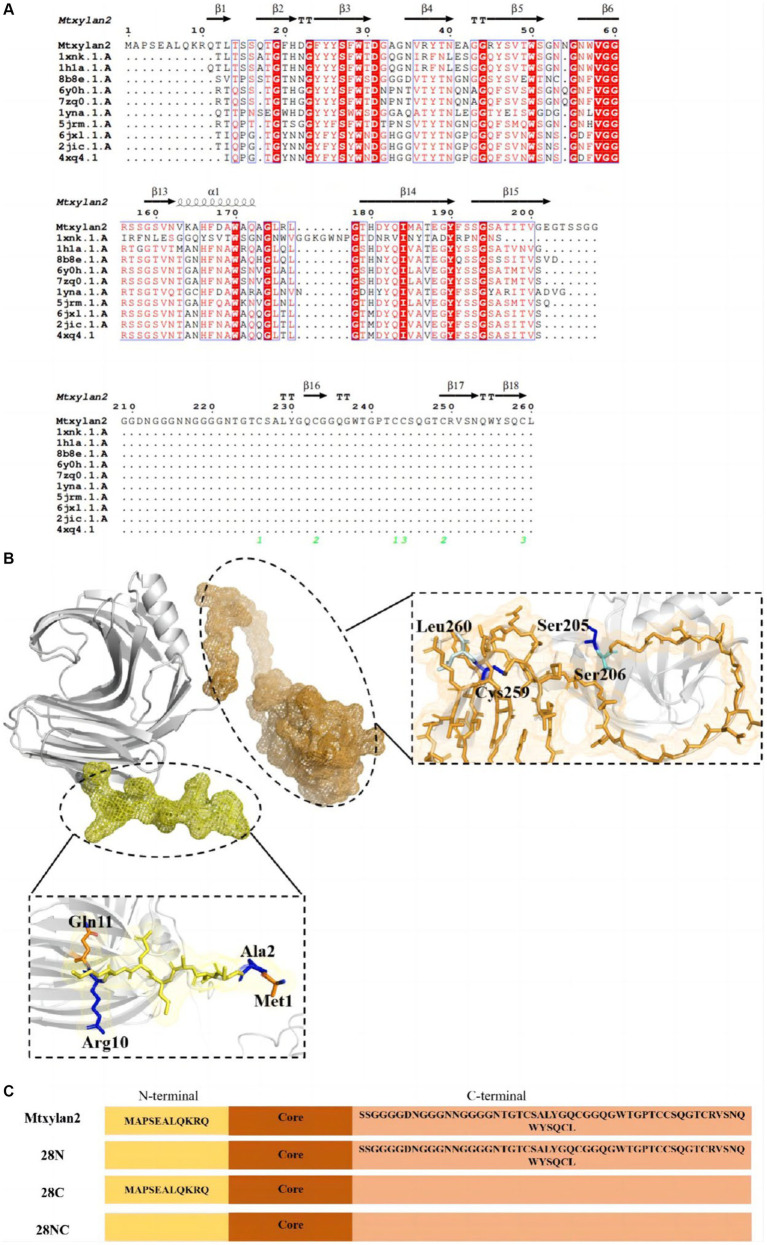
**(A)** Amino acid sequence comparison of the N-terminal sequence of Mtxylan2, 28N, 28C, and 28NC. ESPript software and Swiss Model Web (http://swissmodel.expasy.org/) were used to compare the amino acid sequence of Mtxylan2, 28N, 28C, and 28NC. **(B)** Schematic overview of recombinantly produced derivatives of Mtxylan2. **(C)** The structure of the mutant is based on PDB:G2Q913.1. N-terminal areas are marked in yellow, and C-terminals are marked in orange. Truncated residues M1-G11 to construct mutant 28N, truncated residues 206S-260L to construct mutant 28C, and simultaneously truncated M1-G11 and 206S-260L to construct 28NC.

The results of BLAST analysis showed the homology of the amino acid sequence of Mtxylan2 with Xz-8 and Xyn11B reached 69.20 and 66.05%, respectively. By analyzing Mtxylan2 with more than 10 xylanases via sequence comparison, it was found that Mtxylan2 has abnormal sequences at the N-terminal (1M-11Q) and C-terminal (206S-260L; Figure 6). N-terminal and C-terminal motifs may play specific roles in protein folding and function (Krishna and Englander, 2005), whereas longer N-termini and C-termini may contribute to poorer thermostability of xylanases (Pan et al., 2022), and rational engineering and mutagenesis of the N-terminal and C-terminal regions are frequently used to improve enzyme activity and thermal stability (Kamondi et al., 2008; Liu et al., 2011; Song et al., 2015; Zheng et al., 2016). Liu et al. (2011) found that by truncating 10N-terminal and 1C-terminal disordered residues, the constructed mutant *Aspergillus niger* xylanase showed a significant increase in thermal stability and activity compared to the wild-type xylanase. Pan et al. (2022) constructed six mutants by truncating the C-terminal residues and found that the specific activity of the mutant strains was 24.46–44.10 times higher than that of the wild-type xylanase. These results suggested that the removal of N- and C-terminal abnormal sequences is a viable way to enhance xylanase activity and thermal stability. Thus, truncation of N-terminal residues with C-terminal residues can significantly improve the ability of xylanase to enhance its thermal stability and other properties, and the strategy of terminal mutation can be utilized to modulate the stability of xylanase and enhance its potential for application (Mahanta et al., 2015).

### 3.6 Construction and biochemical analysis of 28N, 28C, and 28NC

**Figure 7:**
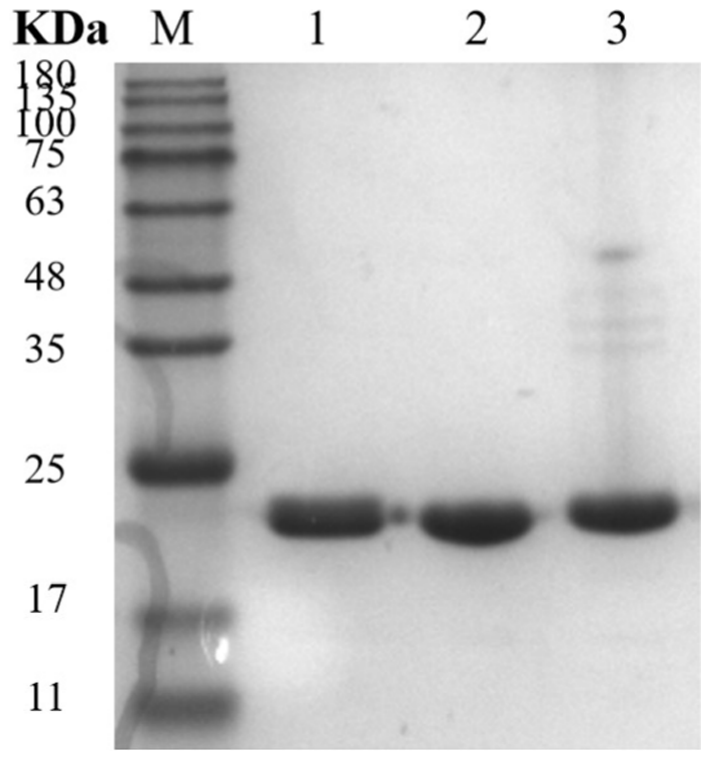
SDS-PAGE analysis of xylanase. Lane M, protein marker; Lane 1, 28N; Lane 2, 28C; and Lane 3, 28NC.

Three mutants, 28N, 28C, and 28NC, were constructed (Figure 7). The catalytic activities of Mtxylan2, 28N, 28C, and 28NC were analyzed using xylan from different sources. Mtxylan2, 28N, 28C, and 28NC showed the highest specific enzyme activity toward beech xylan, followed by birch xylan and oat-spelled xylan. This indicated that Mtxylan2, 28N, 28C, and 28NC had similar substrate preferences (Table 1). Compared to Mtxylan2, 28N, 28C, and 28NC showed 2.4-fold, 4.1-fold, and 9.3-fold higher specific activities for beechwood xylan, 7.1-fold, 26.7-fold, and 10.4-fold higher specific activities for birchwood xylan, and 4.5-fold, 13.4-fold, and 7.9-fold higher specific activities for oat spelling, respectively (Table 1). These results suggested that truncation of the N-terminus, C-terminus, and both the N-terminus and C-terminus of Mtxylan2 enhanced its specific enzyme activity, which was in agreement with the findings of Kang and Ishikawa (2007), Li et al. (2018), Liu et al. (2011) and Pan et al. (2022). The kinetic parameters of the enzymes with beechwood xylan are listed in Table 2. Compared with Mtxylan2, 28C showed a 1.1-fold decrease in substrate affinity (*K*_m_) and a 6.7-fold increase in catalytic efficiency (*k*_cat_/*K*_m_; Table 2).

**Table 2:** Kinetic parameters of Mtxylan2, 28N, 28C, and 28NC.

Enzyme	*V*_max_ (μmol·min^−1^·mg^−1^)	*K*_m_ (mg·mL^−1^)	*k*_cat_ (s^−1^)	*k*_cat_/*K*_m_ (mL·mg^−1^·s^−1^)
Mtxylan2	218.8 ± 5.61	34.60 ± 4.38	106.37 ± 2.73	3.07
28N	781.70 ± 35.62	23.95 ± 2.55	344.34 ± 15.69	14.38
28C	1668.00 ± 97.37	30.27 ± 2.83	620.50 ± 36.22	20.50
28NC	919.10 ± 5.92	25.53 ± 0.65	328.12 ± 2.08	12.85

**Figure 8:**
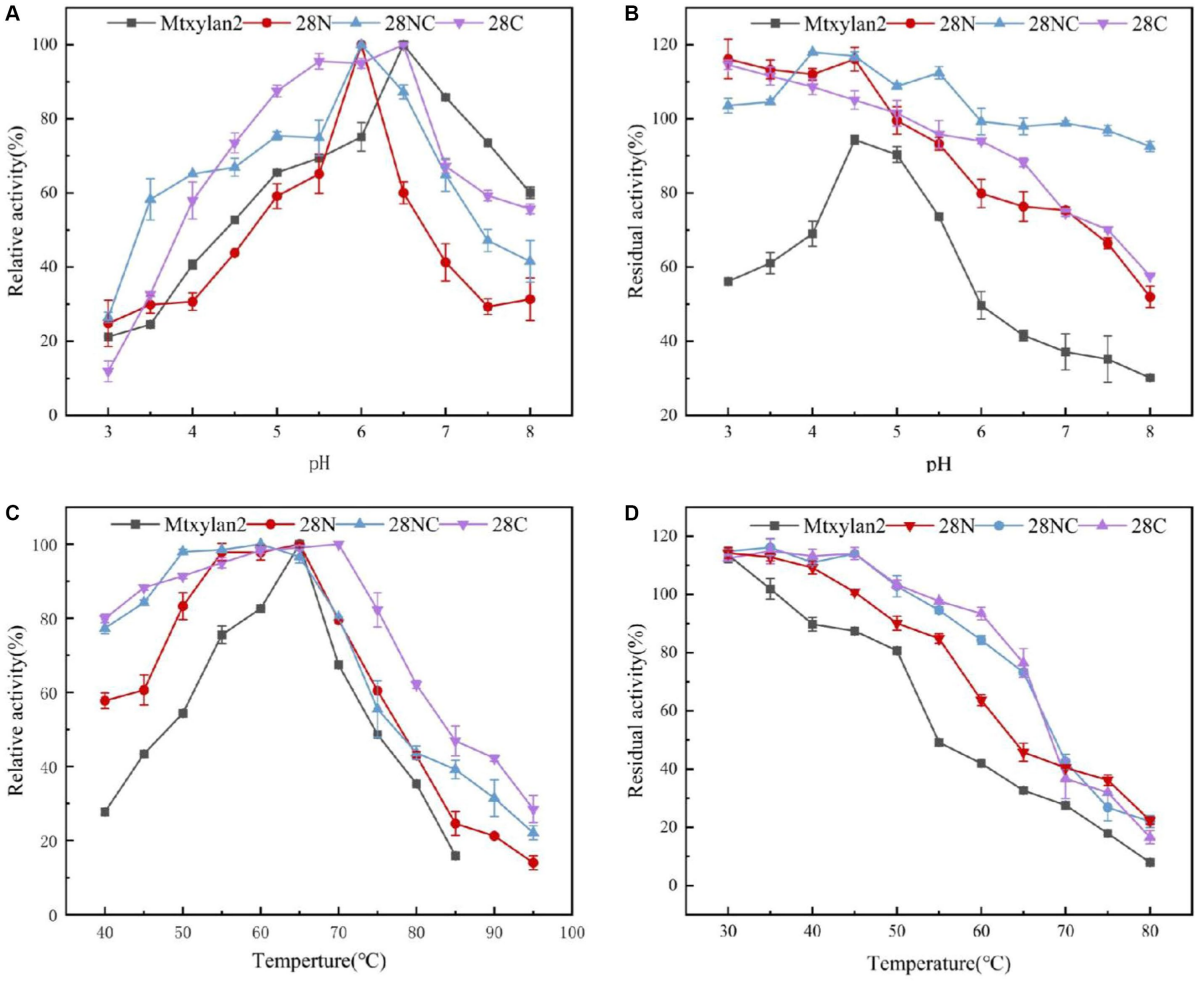
The biochemical characterization of Mtxylan2, 28N, 28C, and 28NC. **(A)** The optimal pH was measured at 50°C for 10 min in different buffers (50 mM) from 3.0 to 8.0; the buffers used were 50 mM citrate buffer (pH 3.0–6.0) and phosphate buffer (pH 6.5–8.0); the highest enzyme activity was used as 100%. **(B)** The pH stability of xylanase was determined in different pH range buffers at 37°C for 30 min, and then the residual activity of the treated enzyme was measured in 50 mM citrate buffer (pH 6.0) at 65°C for 10 min; the activity of untreated xylanase was defined as 100%. **(C)** The optimal temperature was measured in 50 mM citrate buffer (pH 6.5) at different temperatures (40–85°C); the highest enzyme activity was used as 100%. **(D)** Thermostability was determined by incubating at varying temperatures (30–80°C) in 50 mM citrate buffer (pH 6.5) for 30 min; the activity of untreated xylanase was defined as 100%.

The optimum pH, temperature, and thermostability of 28N, 28C, and 28NC were analyzed. The optimal pH of 28N and 28NC was determined to be 6.0 at 37°C, which is 0.5 lower compared to wild-type Mtxylan2, and the optimal pH of 28C was 6.5, the same as Mtxylan2 (Figure 8A). In addition, the pH stability of the xylanases was determined after incubation at pH 6.0 and pH 6.5 for 30 min (Figure 8B). Under acidic pH conditions, 28N, 28C, and 28NC showed better stability than Mtxylan2. After 30 min of incubation at pH 3.0–5.5, 28N, 28C, and 28NC showed more than 80% of their maximum activity, while Mtxylan2 showed <20% of its maximum activity.

**Figure 9:**
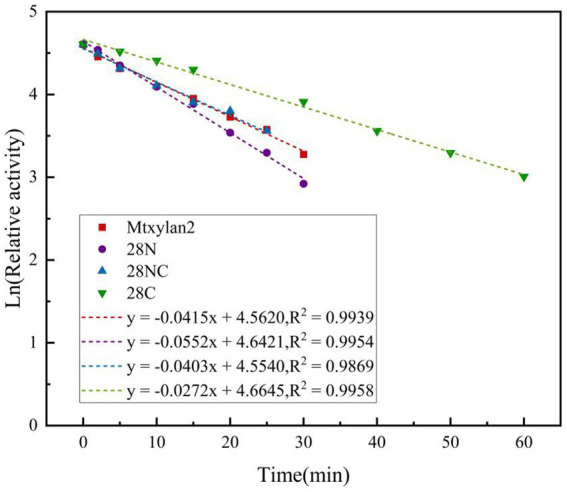
The half-life of the enzyme was determined by incubating at different intervals in 50 mM citrate buffer (pH 6.0 and pH 6.5), and the relative activity was measured at 60°C.

The optimum temperature of the wild-type Mtxylan2 was 65°C. The optimum temperatures of 28N and 28NC were the same as the wild-type Mtxylan2, while the optimum temperature of 28C was increased by 5°C over the wild-type to 70°C (Figure 8C). When held between 50°C and 65°C for 30 min, 28NC and 28C retained more than 80% of their residual activity, and 28N retained about 50% of its activity, whereas wild-type Mtxylan2 retained only 30% of its activity at 65°C (Figure 8D). This is consistent with previous studies. Liu et al. (2011) found that truncating the N-terminal and C-terminal of *Aspergillus niger GH10 xylanase* (Xyn) resulted in an increase in its thermal stability and optimal temperature. In addition, the half-life of Mtxylan2, 28N, 28C, and 28NC at 60°C were 13.1 min, 15 min, 15 min, and 27.7 min, respectively, indicating that 28N, 28C, and 28NC are more thermally stable than Mtxylan2 at 60°C (see Figure 9). This is consistent with the findings of Han et al. (2018). Han et al. (2018) truncated the C-terminus of *Aspergillus oryzae Li-3* (PGUS) and found that the C-terminal mutant had an increased half-life and significantly improved kinetic and thermodynamic stability. These results suggested that truncating the N-terminal, C-terminal, or NC-terminal can change the optimal temperature of xylanase and make it more adaptable to high-temperature industrial production.

### 3.7 Analysis of possible mechanisms for thermal stability of 28N, 28C, and 28NC

The above results showed that the biochemical properties of 28C were differentially enhanced compared to Mtxylan2. The 28C enzyme activity was 9-fold higher than that of Mtxylan2. Moreover, compared with Mtxylan2, the optimum temperature of 28C was increased by 5°C, the half-life at 60°C was prolonged by 14 min, the *K*_m_ value was reduced by 1.1-fold, and *k*_cat_/*K*_m_ was increased by 6.7-fold. 28C has higher catalytic activity and thermal stability compared with other truncated mutants. So we compared and analyzed the RMSD, RMSF, C-terminal hydrogen bonding, and electrostatic potential energy of Mtxylan2 and 28C.

**Figure 10:**
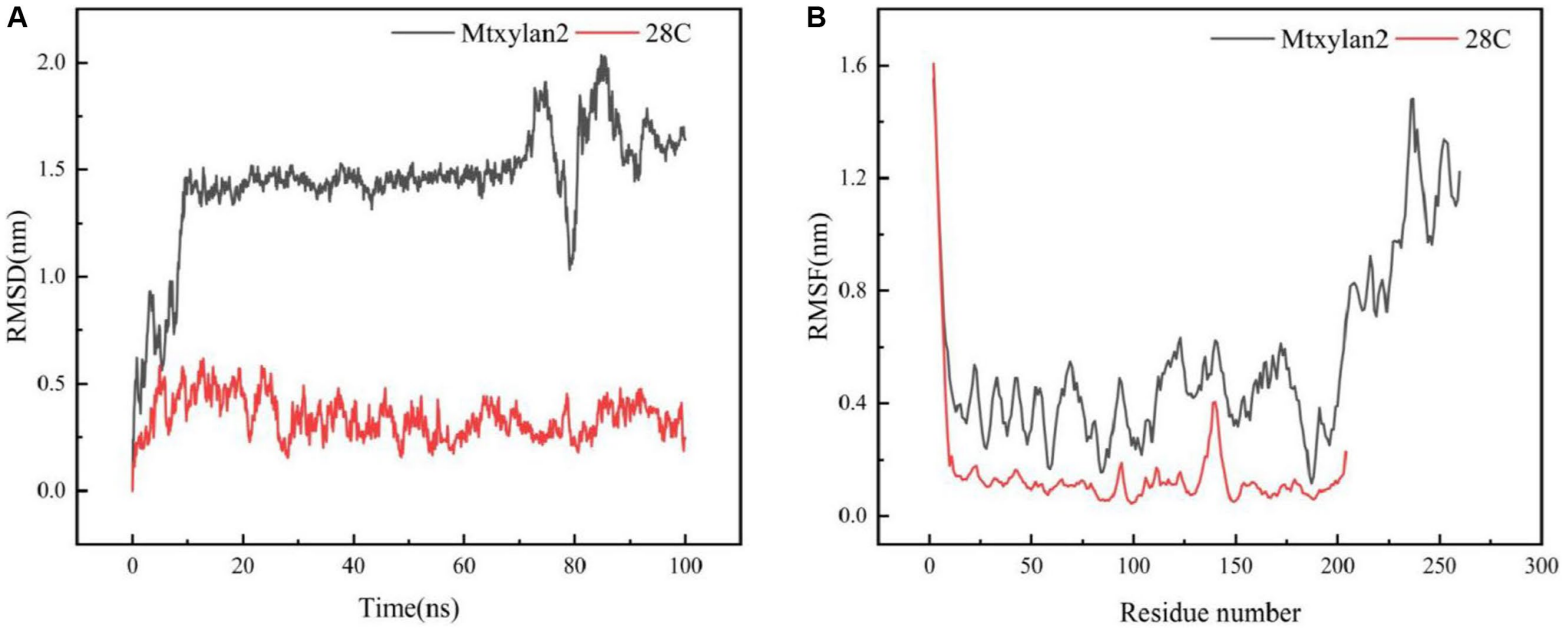
Molecular dynamics simulation of Mtxylan2 and 28C. The RMSD **(A)** and RMSF **(B)** values of Mtxykan2 and 28C at 333 K.

The molecular dynamics of Mtxylan2, 28C at 333 K and 100 ns were simulated using Gromac 4.3.4 software. The RMSD value of 28C was always lower than that of Mtxylan2 at 0–100 ns (Figure 10A), and the RMSF value of Mtxylan2 was higher than that of 28C at 0–300 ns (Figure 10B). Compared with Mtxylan2, the RMSD and RMSF values of 28C were lower, indicating that C-terminal truncation reduced the flexibility of amino acid residues, which resulted in the compression of xylanase structure and improved the conformational stability of the enzyme. Root mean square deviation (RMSD) is an important parameter for estimating protein conformational heat fluctuations, and RMSD reflects the flexibility of each residue in MD simulation. Protein thermal stability was negatively correlated with RMSD and RMSF values (Vucinic et al., 2021). Cayetano-Cruz et al. (2021) found that the highly thermally stable xylanase Xyn11A-N11Y has low RMSD and RMSF values.

**Figure 11:**
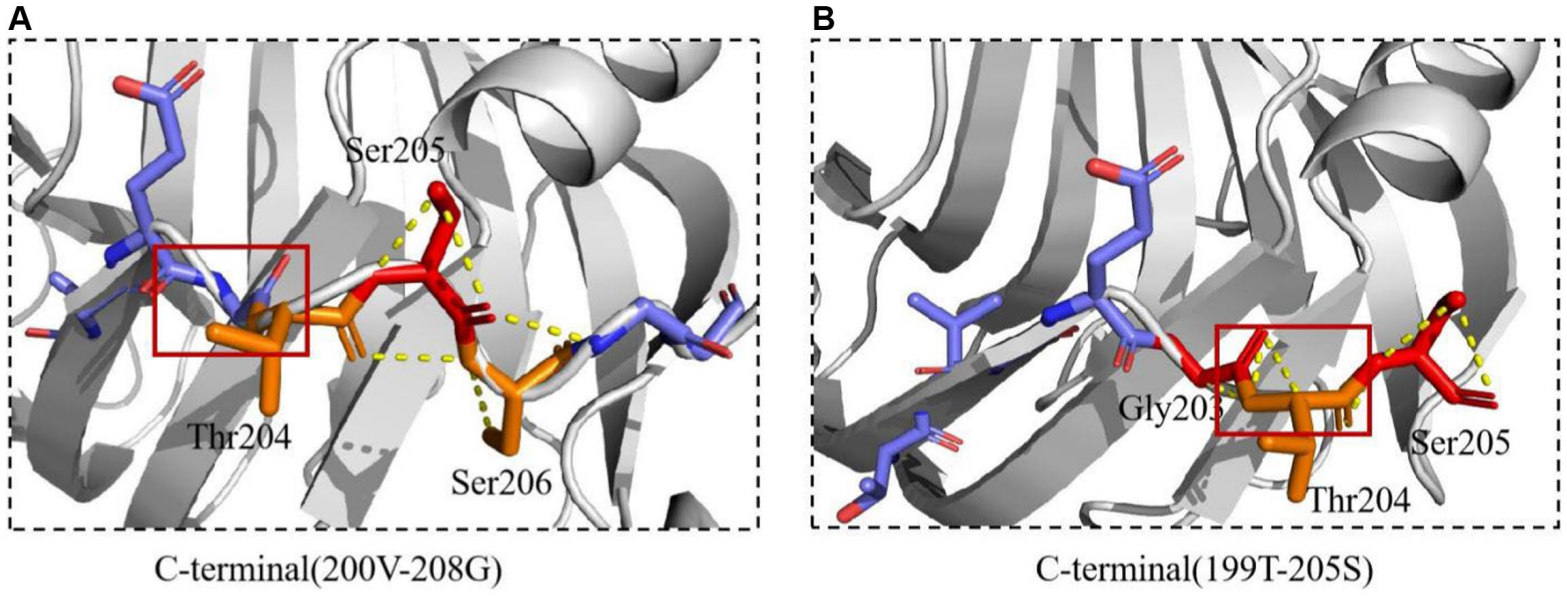
The interactions of xylanase were predicted. **(A)** The interactions of Mtxylan2. **(B)** The interactions of 28C.

The interaction between Mtxylan2 and 28C was compared. A new interaction was found around the C-terminal mutation site (Figure 11). The hydrogen bonding interaction between Mtxylan2 and 28C at Ser206 is altered. In 28C, because of the truncation of the C-terminal Ser206, this results in a hydrogen bond break between the original Ser206, Ser205, and Thr204. Ser205 re-formed new hydrogen bonds with Thr204 and Gly203 (Figure 11). Changes in intramolecular hydrogen bonding interaction forces play an important role in improving the thermal stability of proteins (Vogt et al., 1997). Changes in hydrogen bonding after truncation may be one of the reasons for the increased thermal stability of 28C.

The hydrophobicity of Mtxylan2 and 28C was analyzed. The GRAVY values of Mtxylan2 and 28C are −0.578 and −0.564. 28C has a lower GRAVY value than Mtxylan2, indicating that the C-terminal truncation leads to an increase in the hydrophobicity of 28C. It has been found that the enhancement of the hydrophobic interaction force promotes the effective encapsulation of the hydrophobic core, thus making the molecular structure more compact and stable (Miyazaki et al., 2006; Kim et al., 2012). Increasing the hydrophobic interaction formed by hydrophobic residues can significantly reduce the conformational entropy of proteins, make the protein structure more dense, and lead to lower C-terminal flexibility, thus improving the thermal stability of enzymes (Li et al., 2022). This may be one of the reasons why C-terminal truncation enhances the thermal stability of the enzyme.

**Figure 12:**
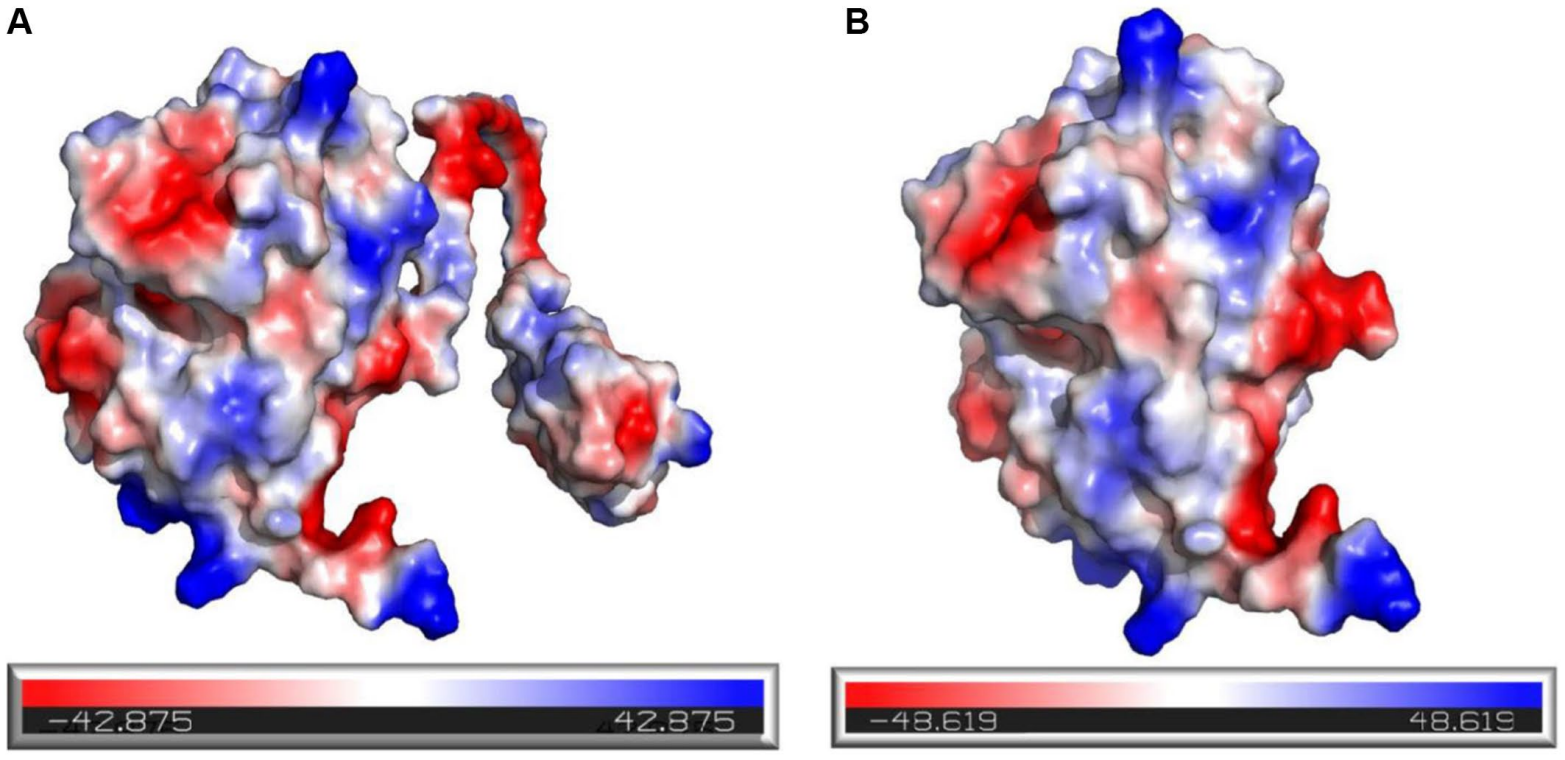
Electrostatic potential energy analysis of Mtxylan2 **(A)** and 28C **(B)**.

In addition, it was observed that the electrostatic potential energy of enzyme 28C after C-terminal truncation was changed from negative to positive (Figure 12A). The Protein Tool was used to calculate the isoelectric point and net charge of Mtxylan2 and 28C as a whole. It was found that the net charge of 28C was changed and the number of positive charges increased to +0.357 (Figure 12B). An increase in positive protein surface charge favors the thermal stability of xylanase (Kumar and Nussinov, 1999). Rutchadaporn et al. (2006) found that increasing the positive surface charge of xylanase from *Aspergillus niger BCC14405* resulted in an enhancement of the thermal stability of xylanase. The increased net surface charge from truncating the C-terminal contributed to the enhanced thermal stability of 28C (Zhou et al., 2019). The above results suggest that the enhanced thermal stability of 28C is partially attributed to changes in hydrogen bonding interaction forces, hydrophobic interactions, and electrostatic potential energy.

### 3.8 Analysis of possible mechanisms for catalytic activity of 28N, 28C, and 28NC

**Figure 13:**
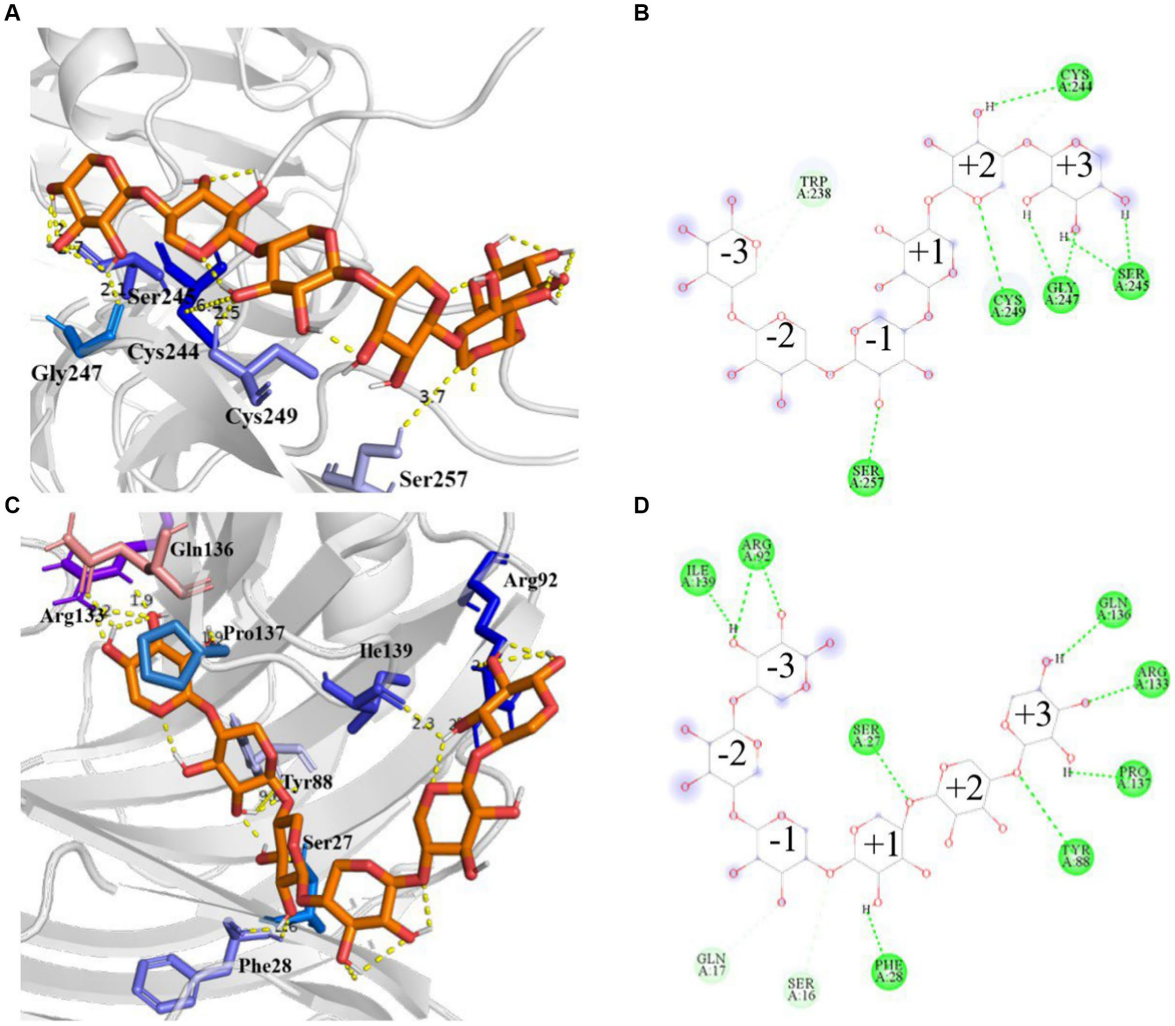
Hydrogen bonding interactions and 2D diagram of the enzyme. Hydrogen bonding interactions of Mtxylan2 **(A)**, 2D diagram **(B)**, hydrogen bonding interactions of 28C **(C)**, and 2D diagram **(D)**.

Molecular docking analyses showed that the hydrogen bonding network patterns between the subsites of Mtxylan2, 28C, and xylohexaose were different. The hydrogen bonding interactions between the subsites of Mtxylan2 with xylohexaose are mainly located at the (+2) and (+3) subsites, whereas those between the subsites of 28C with xylohexaose are mainly located at the (−3) and (+3) subsites (Figures 13A,B). Compared to Mtyxlan2, 28C increased hydrogen bonding interactions between the (+1) subsite and xylohexaose (Figures 13C,D). Interactions formed between the subsites located at the (−3) position and the substrate correlate negatively with product release efficiency (Wu et al., 2018), whereas interactions formed between the subsites located at the (−1) and (+1) positions correlate positively with product release efficiency (Paes et al., 2007; Pollet et al., 2010). Changing the pattern of substrate-subsite interactions accelerates the cycle of catalytic behavior, which in turn increases the catalytic efficiency and catalytic activity of 28C. This is one of the reasons why C-terminal truncation affects the catalytic activity of xylanases.

**Figure 14:**
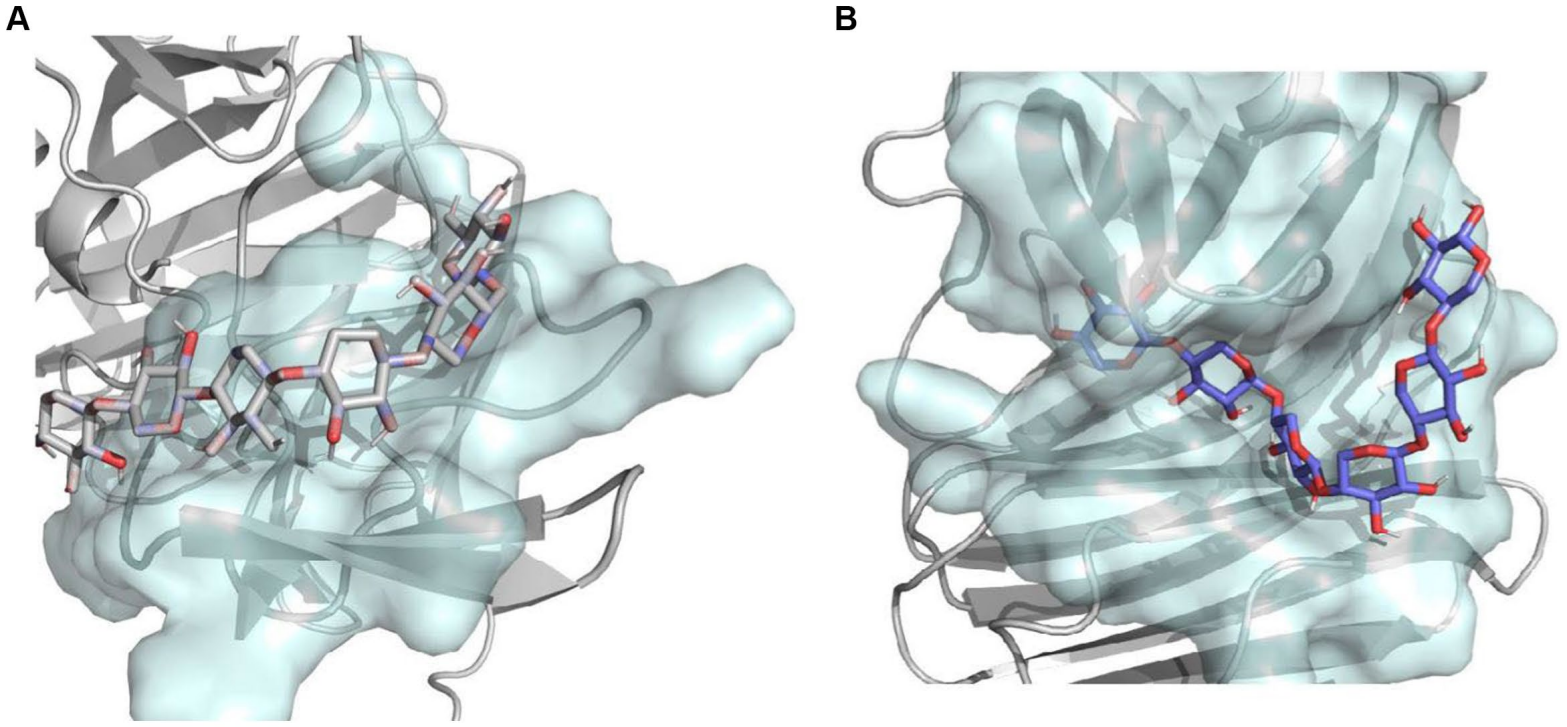
The combined pocket visualization analysis of Mtxylan2 and 28C.

In addition, to better understand the effect of C-terminal truncation on catalytic activity, we calculated the binding pocket volumes of mutants 28C and Mtxylan2. Calculation of the size of the binding pocket of the enzyme-substrate complex revealed that the value of the binding pocket volume of Mtxylan2 was less than that of 28C (Table 3). Visualization of the results using PyMol revealed an increase in the volume of the binding pocket for 28C (Figures 14A,B). The volume change of the protein binding pocket can reflect the alternating changes in protein folding state and stability. The increase in binding pocket volume favors the contact between enzyme and substrate, improves the affinity between enzyme and substrate, and facilitates the improvement of enzyme activity. The larger binding pocket volume is favorable to providing space for enzyme-substrate binding and product release, which leads to a higher specific activity of the enzyme and faster catalytic efficiency (Han et al., 2017; Tian et al., 2017; Xing et al., 2021; Chen et al., 2022). The above results suggested that the enhanced thermal stability of 28C was partly due to the alteration of interactions formed between the subunit and substrate as well as the alteration of the binding pocket volume.

**Table 3:** Solvent accessible surface area of Mtxylan2 and 28C.

Enzyme	Volume (Å^3^)
Mtxylan2	899.07
28C	1046.85

## 4 Conclusion

In this study, GH11 xylanase Mtxylan2 with potential thermotolerant activity was obtained by bioinformatics analysis based on the transcriptome data of thermophilic destructive filamentous bacterium ATCC42464. The optimum pH, temperature, and enzyme activity of Mtxyaln2 were 6.0, 65°C, and 104.67 U/mg, respectively. To improve the thermal stability and catalytic activity of Mtxylan2, we performed N-terminal and C-terminal truncation and constructed three mutants, 28N, 28C, and 28NC. 28C was found to have higher catalytic activity and thermostability than 28N and 28NC. Compared to Mtxylan2, 28C exhibited enhanced pH stability under acidic conditions, enhanced thermal stability at 55–75°C, and improved catalytic activity. Computer simulation analyses showed that new hydrogen bonds and electrostatic interactions were formed at the C-terminus of 28C, resulting in reduced flexibility of the C-terminus, increased binding pocket size, and changes in the hydrogen bonding network between the subsites located in the cleft and the substrate. These findings provided possible ideas for improving the thermostability and the catalytic properties of GH11 xylanase.

## Data availability statement

The datasets presented in this study can be found in online repositories. The names of the repository/repositories and accession number(s) can be found in the article/Supplementary material.

## Author contributions

YY: Writing – original draft, Writing – review & editing. CZ: Writing – original draft, Writing – review & editing. HL: Investigation, Software, Writing – review & editing. QW: Data curation, Methodology, Supervision, Writing – review & editing. YW: Formal analysis, Project administration, Validation, Writing – review & editing. WL: Conceptualization, Formal analysis, Project administration, Validation, Writing – review & editing. XL: Funding acquisition, Resources, Visualization, Writing – review & editing.

## Footnotes

^1^ https://saves.mbi.ucla.edu/

^2^ http://www.cbs.dtu.dk/services/SignalP/
