# Supplementary material for: Improvement of thermostability and catalytic efficiency of xylanase from Myceliophthora thermophilar by N-terminal and C-terminal truncation
Source: Front Microbiol. 2024 Apr 10;15:1385329. doi: 10.3389/fmicb.2024.1385329 (PMC11039872; doi:10.3389/fmicb.2024.1385329)
Supplement: Supplementary file 1 [file Data_Sheet_1.docx]

Supplementary material

Improvement of thermostability and catalytic efficiency of xylanase from *Myceliophthora thermophilar* by N-terminal and C-terminal truncation

Yue Yang ^1,2†^, Chengnan Zhang ^3†^, Hongyun Lu ^1,2^，QiuHua Wu ^1,2^，Yanfang Wu^1,2^, Weiwei Li^1,2^ and Xiuting Li ^1,2*^

^1^Key Laboratory of Geriatric Nutrition and Health (Beijing Technology and Business University), Ministry of Education, Beijing, 100048, PR China

^2^Beijing Engineering and Technology Research Center of Food Additives, Beijing Technology & Business University (BTBU), Beijing, 100048, PR China

^3^Department of Exercise Biochemistry, Exercise Science School, Beijing Sport University, Beijing, 100048, PR China

† These authors contribute equally to this work

#These authors contribute equally to this work

# Supplementary Tables

**Table S1.** The primers used in experiments.

| Primers | Sequences (5’ - 3’) |
| --- | --- |
| 1S11f (*Nco*I) | **CATGCCATG** ATGGCGCCAAGCGAAGCGCTG |
| 1S11r (*Xho*I) | **CCGCTCGAG** CAGGCACTGGCTATACCACTG |
| 28Nf(*Nco*I) | **CATGCCATG** ATGGCGCCAAGCGAAGCGCTG |
| 28Nr(*Xho*I) | **CCGCTCGAG** CAGGCACTGGCTATACCACTG |
| 28Cf(*Nco*I) | **CATGCCATG** ATGGCGCCAAGCGAAGCGCTG |
| 28Cr(*Xho*I) | **CCGCTCGAG** GGTACCTTCGCCCACGGTAAT |
| 28NCf(*Nco*I) | **CATGCCATG** ACTCTGACCAGCAGCCAGACCGGCTTT |
| 28NCr(*Xho*I) | **CCGCTCGAG** GGTACCTTCGCCCACGGTAAT |

**Table S2.** Functional annotation of key genes.

| Key Genes | GO Function | Similar Genes ID |
| --- | --- | --- |
| MYCTH_2298452 | Uncharacterized proteins of thermophilic destructive filamentous bacteria | XM_003660259.1 |
| MYCTH_2297820 | Transaldolase (enzyme) | XM_008100829.1 |
| MYCTH_2298386 | Glucose-6-phosphate isomerase | XM_001223629.1 |
| MYCTH_2302660 | Transferase (enzyme) | XM_060436890.1 |
| MYCTH_100068 | Glycoside hydrolase family 11 Xylanase | XP_003662402.1 |
| MYCTH_2301113 | 3-Hydroxyacyl-coenzyme A dehydrogenase | XM_060441406.1 |
| MYCTH_2310435 | Acyl Coenzyme A Dehydrogenase | XM_009850884.1 |

| **Table S3.** Comparison of Mtxylan2 and 28C with the reported GH11 xylanases | | | | | | | |
| --- | --- | --- | --- | --- | --- | --- | --- |
| Xylanase | Organism | optimum pH | T_opt_ (℃) | Thermostability | Specific activity (U/mg) | half-life | References |
| XynZF-2 | Aspergillus niger XZ-3S | 5.0 | 40 | at 40°C for 60  min, 44.36% | - | 45°C，7 min | (C.-Y. Zhou, Li, Wang, Zhu, & Kang, 2016) |
| Xyn11NX | Nesterenkonia xinjiangensis CCTCC AA001025 | 7.0 | 55 | at 60 °C for 1 h，80% | 2158 | - | (Kui et al., 2010) |
| RrXyn11AL | Chytridiomycetous fungus, *Rhizophlyctis rosea* | 7.0 | 40 | at 70 °C for 1 h，65% | 210.7 | - | (Huang et al., 2019) |
| XynSW1 | Thermotolerant Streptomyces sp. SWU10 | 5.0 | 40 | at 50 °C ,100 % | 13.3 | - | (Sukhumsirichart et al., 2014) |
| Tnap_0700 | [*Thermotoga naphthophila*](https://webofscience.clarivate.cn/wos/alldb/full-record/WOS:000500534800016) | 7.0 | 37 | at 80 °C for 2 h，40.50% | 144.78 | - | (Hamid & Aftab, 2019) |
| Xyl8 | Myceliophthora thermophila C1 | 6.5 | 50 | at 20-65°C，≥50% | 170.6 | - | (van Gool et al., 2013) |
| MYCTH_56237 | Myceliophthora thermophila | 6.0 | 60 | at 60°C,60% | 1533.7 | - | (Basit et al., 2018) |
| XynFCB | Thermoanaerobacterium saccharolyticum NTOU1 | 6.4 | 63 | at 65 °C for 90 min，40% | 91 | 65°C,55 min | (Hung et al., 2011) |
| xynA | Bacillus pumilus ARA | 6.6 | 50 | at 60°C for 1 h，≥50% | - | 60°C,1 h | (Qu & Shao, 2011) |
| XynCDBFV | Fungal GH11 xylanase | - | 60 | at 60℃ for 1 h，20.94% | - | - | (Han et al., 2019) |
| xynA | Trichoderma sp. SC9 | 6.0 | 42.5 | at 60 °C,80% | 676.8 | 45 °C,23.9 min | (P. Zhou, Zhu, Yan, Katrolia, & Jiang, 2011) |
| XynS20E | Neocallimastix patriciarum the ruminal fungus | 5.8 | 49 | - | 873.1 | - | (Pai et al., 2010) |
| thxyn11A | Thermobifida halotolerans strain YIM 90462(T) | 9.0 | 70 | at 70 °C for 30 min,90 % | - | - | (Zhang et al., 2012) |
| XynLC9 | Bacillus subtilis | 7.0 | 60 | at 50℃ for 4 h,62.2% | 1570.0 | - | (Wang et al., 2021) |
| Xyn11A | Paenibacillus curdlanolyticus B-6 | 6.0 | 60 | - | 89.7 | - | (Sermsathanaswadi et al., 2014) |
| Srxyn | Streptomyce rochei L10904 | 6.0 | 70 | at 70 °C for 60 min,25% | 58.16 | - | (Li et al., 2017) |
| Mtxylan2 | Myceliophthora thermophila ATCC42464 | 6.5 | 65 | at 65°C for 30 min，30% | 104.67 | 60℃，13.2 min | Present study |
| 28C | Myceliophthora thermophila ATCC42464 | 6.5 | 70 | at 65°C for 30 min，80% | 973.74 | 60℃，27.2 min | Present study |

Reference:

Basit, A., Liu, J., Miao, T., Zheng, F., Rahim, K., Lou, H., & Jiang, W. (2018). Characterization of Two Endo-β-1, 4-Xylanases from *Myceliophthora thermophila* and Their Saccharification Efficiencies, Synergistic with Commercial Cellulase. *Frontiers in Microbiology, 9*. doi:10.3389/fmicb.2018.00233.

Hamid, A., & Aftab, M. N. (2019). Cloning, Purification, and Characterization of Recombinant Thermostable β-Xylanase Tnap_0700 from *Thermotoga naphthophila*. *Applied Biochemistry and Biotechnology, 189*(4), 1274-1290. doi:10.1007/s12010-019-03068-0.

Han, N., Ma, Y., Mu, Y., Tang, X., Li, J., & Huang, Z. (2019). Enhancing thermal tolerance of a fungal GH11 xylanase guided by B-factor analysis and multiple sequence alignment. *Enzyme and Microbial Technology, 131*. doi:10.1016/j.enzmictec.2019.109422.

Huang, Y., Zheng, X., Pilgaard, B., Holck, J., Muschiol, J., Li, S., & Lange, L. (2019). Identification and characterization of GH11 xylanase and GH43 xylosidase from the chytridiomycetous fungus, *Rhizophlyctis rosea*. *Applied Microbiology and Biotechnology, 103*(2), 777-791. doi:10.1007/s00253-018-9431-5.

Hung, K.-S., Liu, S.-M., Tzou, W.-S., Lin, F.-P., Pan, C.-L., Fang, T.-Y., . . . Tang, S.-J. (2011). Characterization of a novel GH10 thermostable, halophilic xylanase from the marine bacterium *Thermoanaerobacterium saccharolyticum* NTOU1. *Process Biochemistry, 46*(6), 1257-1263. doi:10.1016/j.procbio.2011.02.009.

Kui, H., Luo, H., Shi, P., Bai, Y., Yuan, T., Wang, Y., . . . Yao, B. (2010). Gene Cloning, Expression, and Characterization of a Thermostable Xylanase from *Nesterenkonia xinjiangensis* CCTCC AA001025. *Applied Biochemistry and Biotechnology, 162*(4), 953-965. doi:10.1007/s12010-009-8815-5.

Li, Q., Sun, B., Jia, H., Hou, J., Yang, R., Xiong, K., . . . Li, X. (2017). Engineering a xylanase from Streptomyce rochei L10904 by mutation to improve its catalytic characteristics. *International Journal of Biological Macromolecules, 101*, 366-372. doi:<https://doi.org/10.1016/j.ijbiomac.2017.03.135>.

Pai, C.-K., Wu, Z.-Y., Chen, M.-J., Zeng, Y.-F., Chen, J.-W., Duan, C.-H., . . . Liu, J.-R. (2010). Molecular cloning and characterization of a bifunctional xylanolytic enzyme from Neocallimastix patriciarum. *Applied Microbiology and Biotechnology, 85*(5), 1451-1462. doi:10.1007/s00253-009-2175-5.

Qu, W., & Shao, W. (2011). Cloning, expression and characterization of glycoside hydrolase family 11 endoxylanase from Bacillus pumilus ARA. *Biotechnology Letters, 33*(7), 1407-1416. doi:10.1007/s10529-011-0568-x.

Sermsathanaswadi, J., Pianwanit, S., Pason, P., Waeonukul, R., Tachaapaikoon, C., Ratanakhanokchai, K., . . . Kosugi, A. (2014). The C-terminal region of xylanase domain in Xyn11A from *Paenibacillus curdlanolyticus* B-6 plays an important role in structural stability. *Applied Microbiology and Biotechnology, 98*(19), 8223-8233. doi:10.1007/s00253-014-5748-x.

Sukhumsirichart, W., Deesukon, W., Kawakami, T., Matsumoto, S., Seesom, W., & Sakamoto, T. (2014). Expression and Characterization of Recombinant GH11 Xylanase from *Thermotoleran*t *Streptomyce*s sp SWU10. *Applied Biochemistry and Biotechnology, 172*(1), 436-446. doi:10.1007/s12010-013-0508-4.

van Gool, M. P., van Muiswinkel, G. C. J., Hinz, S. W. A., Schols, H. A., Sinitsyn, A. P., & Gruppen, H. (2013). Two novel GH11 endo-xylanases from *Myceliophthora thermophila* C1 act differently toward soluble and insoluble xylans. *Enzyme and Microbial Technology, 53*(1), 25-32. doi:10.1016/j.enzmictec.2013.03.019.

Wang, L., Cao, K., Pedroso, M. M., Wu, B., Gao, Z., He, B., & Schenk, G. (2021). Sequence- and structure-guided improvement of the catalytic performance of a GH11 family xylanase from Bacillus subtilis. *Journal of Biological Chemistry, 297*(5), 101262. doi:<https://doi.org/10.1016/j.jbc.2021.101262>.

Zhang, F., Chen, J.-J., Ren, W.-Z., Lin, L.-B., Zhou, Y., Zhi, X.-Y., . . . Li, W.-J. (2012). Cloning, expression, and characterization of an alkaline thermostable GH11 xylanase from *Thermobifida halotolerans* YIM 90462T. *Journal of Industrial Microbiology & Biotechnology, 39*(8), 1109-1116. doi:10.1007/s10295-012-1119-8.

Zhou, C.-Y., Li, T.-B., Wang, Y.-T., Zhu, X.-S., & Kang, J. (2016). Exploration of a N-terminal disulfide bridge to improve the thermostability of a GH11 xylanase from *Aspergillus nige*r. *Journal of General and Applied Microbiology, 62*(2), 83-89. doi:10.2323/jgam.62.83.

Zhou, P., Zhu, H., Yan, Q., Katrolia, P., & Jiang, Z. (2011). Purification and Properties of a Psychrotrophic Trichoderma sp Xylanase and its Gene Sequence. *Applied Biochemistry and Biotechnology, 164*(6), 944-956. doi:10.1007/s12010-011-9186-2.
